# Tumor Necrosis Factor-α, Matrix-Metalloproteinases 8 and 9 Levels in the Saliva Are Associated with Increased Hemoglobin A1c in Type 1 Diabetes Subjects

**DOI:** 10.1371/journal.pone.0125320

**Published:** 2015-04-27

**Authors:** Melanie N. Kuehl, Henry Rodriguez, Brant R. Burkhardt, Amy C. Alman

**Affiliations:** 1 Department of Cell Biology, Microbiology and Molecular Biology, University of South Florida, Tampa, Florida, the United States of America; 2 Diabetes Center, College of Medicine, University of South Florida, Tampa, Florida, the United States of America; 3 Department of Epidemiology and Biostatistics, College of Public Health, University of South Florida, Tampa, Florida, United States of America; University of Florida, UNITED STATES

## Abstract

**Background:**

Type 1 diabetes (T1D) is an autoimmune disease resulting in the targeted destruction of pancreatic β-cells and permanent loss of insulin production. Proper glucose management results in better clinical outcomes for T1D and provides a strong rationale to identify non-invasive biomarkers indicative or predictive of glycemic control. Therefore, we investigated the association of salivary inflammation with HbA_1c_ in a T1D cohort.

**Methods:**

Unstimulated saliva was collected from 144 subjects with T1D at the USF Diabetes Center. BMI, duration of diabetes, and HbA_1c_ were recorded during clinical visit. Levels of interleukin (IL)-1β, -6, -8, -10, IFN-γ, TNF-α, MMP-3, -8, and -9 were measured using multiplexing immunoassay analysis. To account for smoking status, salivary cotinine levels were also determined.

**Results:**

Multiple linear (HbA_1c_) and logistic (self-reported gingival condition) regression analyses were performed to examine the relationships between the Principal Component Analysis (PCA) components and HbA_1c_ and gingival condition (adjusted for age, duration of diabetes, BMI, and sex; model for HbA_1c_ also adjusted for gingival condition and model for gingival condition also adjusted for HbA_1c_). PCA components 1 (MMP-8 and MMP-9) and 3 (TNF-α) were significantly associated with HbA_1c_ (β = 0.28 ±0.14, p = 0.045; β = 0.31 ±0.14, p = 0.029), while PCA component 2 (IL-6, IL-1β, and IL-8) was significantly associated with gingival condition (OR 1.60 95% CI 1.09–2.34, p = 0.016). In general, increased salivary inflammatory burden is associated with decreased glycemic control and self-reported gingival condition.

**Conclusions:**

The saliva may represent a useful reservoir of novel noninvasive inflammatory biomarkers predictive of the progression and control of T1D.

## Introduction

Periodontitis impacts as much as 47% of the U.S. population and is a significant cause for tooth loss in adults [1]. This destructive process is driven by bacterial infections that colonize the tooth root surface [2]. Due to this pathogenic event, immunological mediators are activated and various metabolic byproducts such as cytokines, chemokines and tissue-destructive enzymes such as matrix-metalloproteinases (MMPs) are released [3]. Spillover of these immunological mediators into the general circulation is thought to play a role in the development and exacerbation of systemic diseases, particularly poorly controlled diabetes, whereby a bi-directional relationship between periodontal disease and glycemic control has been suggested [4, 5]. Type 1 diabetes (T1D) is a highly complex polygenic autoimmune disease resulting in the loss of pancreatic β-cells and absence of insulin production [6]. While the relationship between periodontal disease and glycemic control has been demonstrated in T1D [7], the association between oral immunological mediators and glycemic control in T1D is not well understood and has not been precisely measured. The overall suspected relationship between periodontal disease and glycemic control provides a strong rationale for our central hypothesis that increased inflammatory burden and quantitative biomarkers of periodontal disease will be associated with decreased glycemic control. To our knowledge, this has never been evaluated in a T1D cohort.

Saliva is a clear mucoserous exocrine derived liquid containing a mixture of secretions from the submandibular, parotid, sublingual and minor glands that provides a representation of overall health status and oral inflammatory burden [8–10]. Saliva can be obtained noninvasively, safely and economically with minimal processing and required training by personnel. Inflammatory molecules within the saliva are derived from the periodontium via influx of gingival crevicular fluid (GCF) and from the mucosa [11]. This bio-collection serves as a highly accessible and useful general measurement of oral inflammatory and periodontal burden. Despite the tremendous potential and utility of the saliva for the examination of biomarkers related to systemic disease, limited studies have been conducted in understanding and evaluating the salivary inflammatory burden specifically in T1D [12–14]. At present, numerous potential surrogate measures of existing periodontal disease and oral health have been identified and include cytokines and MMPs such as interleukin-1β (IL-1β), tumor necrosis factor (TNF)-α, and matrix metalloproteinase (MMP)-8 [10, 15–17]. The utility of these biomarkers has been demonstrated in terms of association with decreased oral health but there are currently no published reports to our knowledge that have examined the association between oral inflammation and levels of HbA1c within T1D.

To address this, we conducted an original study to examine the association between salivary inflammatory burden with glycemic control (HbA_1c_) and self-reported gingival condition in adult T1D subjects recruited from the University of South Florida Diabetes Center.

## Materials and Methods

### Participants

A cross-sectional observation study of 150 T1D patients consecutively recruited from the Diabetes Center at the University of South Florida, aged 18 or older, was conducted to examine the association between salivary inflammation and glycemic control. Subjects were recruited during regularly scheduled clinic visits. Of the 150 that were enrolled and that provided an unstimulated whole saliva sample (described below), 6 subjects were excluded from this analysis due to their saliva being very viscous and/or evidently contaminated with blood. Nine additional subjects were excluded from the MMP analysis due to inadequate quantity of saliva. Only 2 subjects approached refused to participate in the study on the basis of overall apprehension of salivary collection. Since there are no published reports of the relationship between HbA1C and salivary cytokine levels in T1D, we have had to base sample size calculations on data from an independent cohort study of adult T1D that used serum measurements. The independent cohort we used did not have measurements for all of the inflammatory cytokines that we are proposing to measure, and due to the possibility that individual inflammatory markers may act on several different pathways to arrive at the same outcome, we based the sample size calculation on a measure of inflammatory burden. We would have a power of >80% to detect a difference in sample means of 0.29 in the inflammatory burden variable using a dichotomization of subjects on HbA1C of ≥ mean vs. < mean. The study was reviewed and approved by the University of South Florida Institutional Review Board. All participants provided informed written consent prior to participation in the study.

### Clinical Data Collection

All enrolled patients in the study completed the oral health questionnaire. This questionnaire consisted of two questions regarding gingival condition: 1) Compared to others your age, how would you rate the current condition of your gums: poor, fair, good, excellent, and 2) Do you have a loose tooth? These questions were previously shown to be correlated with clinically determined periodontal health [18]. Additional information obtained from the patient chart at the time of the clinic visit included body mass index (BMI), duration of diabetes, age, sex, race, and glycated hemoglobin (HbA_1c_), a measure of glycemic control

### Saliva Collection

Each participant passively drooled in a 2 mL collection tube with an attached salivary collection aid (Saliva Biol LLC No. 61/524096 patent pending). Approximately 1 to 1.5 ml of saliva was collected from each subject. Immediately upon collection a protease and phosphatase inhibitor cocktail (EDTA-free Thermo Scientific Halt, Thermos Fisher Scientific, Rockford, IL, USA) was added at 1X to inhibit proteolysis. The sample was centrifuged for 5 minutes at 5500 rpm in a refrigerated centrifuge set at -10°C. The saliva sample was then distributed into 100 μL aliquots and stored immediately at -80°C until multiplexing analysis. Commercially received saliva (Innovative Research, Novi, MI, USA) was also aliquoted and stored frozen to be later used within each multiplexing batch for the purpose of quality control.

### Cytokine Analysis

Cytokine levels were determined using a multiplexed bead immunoassay and measured with a Luminex MAGPIX instrument (Luminex, Austin, TX, USA). Six cytokines: IL-1β, IL-6, IL-8, IL-10, TNF-α, and IFN-γ was measured using the high sensitivity human cytokine magnetic bead assay (EMD Millipore, Cat No. HSCYTMAG-60SK-06, Billerica, MA, USA) following manufacturer instructions. Each assay was analyzed on the Luminex MAGPIX instrument to measure inflammatory concentrations followed by a 5-parameter logistic curve-fitting method from a standard curve of each respective analyte. Saliva samples were normalized by equivalent volume (155 μl) and examined in duplicate per each assay run. The high sensitivity human cytokine magnetic bead kit provides a minimum detectable concentration of IL-1β (0.06 pg/mL), IL-6 (0.20 pg/mL), IL-8 (0.05 pg/mL), IL-10 (0.48 pg/mL), TNF-α (0.07 pg/mL), and IFN-γ (0.18 pg/mL). Quality control samples of non-T1D saliva were included in each assay run to account for any potential intra or inter-assay variability to ensure that % CV did not exceed 3%. Concentration was calculated by the StatLIA Immunoassay Analysis software (Brendan Technologies), by measuring the true limits of detection for an assay by mathematically determining what the empirical Minimum Detectable Concentration (MinDC) would be if an infinite number of standard concentrations were run for the assay under the same conditions. Measurements were performed in triplicate.

### MMP Analysis

Human matrix metalloproteinase (MMP) levels were determined using a multiplexed bead immunoassay. Three MMPs: MMP-3, MMP-8, and MMP-9 were measured using the high sensitivity human MMP Base magnetic bead assay (R&D systems Inc, Catalog number LMPM000, Minneapolis, MN, USA). The plate was analyzed on the Luminex MAGPIX instrument as described above for determination of cytokine concentration. Measurements were performed in triplicate.

### Cotinine Analysis

Salivary cotinine levels were measured utilizing a commercial ELISA (Salimetrics, Catalog Number 1–2002, Carlsbad, CA, USA) according to manufacturer’s instructions. Cotinine levels (ng/ml) were determined from 20 μl of saliva and measured in duplicate. Due to saliva volume limitations, cotinine levels were determined for 95 of the 144 subjects. Results are reported as means and standard deviations.

### Statistical Analysis

Data are presented as means and standard deviations for continuous variables and as the number of subjects and percent for categorical data. Distributions of all of the cytokines and MMPs were found to be skewed and were log transformed for all analyses. For these variables, the data are presented as the geometric mean and interquartile range. All cytokines were adequately measured within the linear range of the generated standard curve with the exception of IFN-γ which was only measured in 2.1% of all salivary samples analyzed and therefore excluded from further analyses. Cotinine-derived smoking status was determined using a cutoff value of 15 ng/ml [19, 20].

Principal components analysis (PCA) with orthogonal rotation was used to produce linear components of the cytokine and MMP variables with shared variance. PCA is a variable reduction technique used to account for redundancy between variables by producing uncorrelated components that account for a meaningful amount of the variance contained in the original set of variables but that can be used simultaneously in a regression analysis. Components may be thought of as independent constructs represented by the markers that load highly on that component. Interpretability of the final solution was a major determinant of the minimum number of components to be used in the analyses. Individual cytokines were considered to load highly on a given component with factor loads >0.6. Factor loads indicate the correlation of individual markers on each component. Multiple linear (HbA_1c_) and logistic (gingival condition) regression analyses were performed to examine the relationships between the PCA components and HbA_1c_ and gingival condition. Both the linear (HbA1c) and logistic (gingival condition) models were adjusted for age, sex, duration of diabetes, and BMI. The HbA1c model was additionally adjusted for gingival condition and the gingival condition model was additionally adjusted for HbA1c. Cotinine-derived smoking status, race, and time of saliva collection were evaluated as potential confounders and not found to alter the parameter estimates for the PCA components in either model by more than 10%, so these were not retained in the final models. All data analyses were performed using SAS/STAT 9.3 software (SAS Institute Inc., Cary, NC).

## Results

### Study design and concentrations of cytokines and MMPs in saliva

The overall characteristics of the subject population are shown in Table 1. The mean age of subjects in this study population was 35.8 (±16.5) years. The study population had slightly more females than males (59% vs 41%) and was predominantly white (79.2%). The mean duration of diabetes was 18.4 (±12.9) years, with a mean HbA_1c_ of 8.3 (±1.7), and a mean BMI of 27.4 (±6.3). Cotinine values indicated that 18 (19.0%) were current smokers. The geometric means of the analytes ranged from 0.25 pg/ml for TNF-α to 305.0 ng/ml for MMP-9.

**Table 1:** Subject characteristics (n = 144).

**Characteristics**	
Age (years)^*^	35.8 ±16.5
Male (n)^†^	59 (41.0)
Race/ethnicity (n)^†^	
White	114 (79.2)
Black	13 (9.0)
Hispanic	12 (8.3)
Other	5 (3.5)
Duration of diabetes (years)^*^	18.4 ±12.9
HbA1c (%)^*^	8.3 ±1.7
BMI (kg/m^2^)^*^	27.4 ±6.3
BMI category (n)^†^	
Underweight (<18.5 kg/m^2^)	5 (3.5)
Normal (18.5-<25 kg/m^2^)	52 (36.1)
Overweight (25-<30 kg/m^2^)	51 (35.4)
Obese (≥30 kg/m^2^)	36 (25.0)
Cotinine (ng/ml)^‡^ ^,^ ^§^	1.1 (0.3–1.7)
Current smoker (n; cotinine > 15 ng/ml)^†^ ^,^ ^§^	18 (19.0)
Time of day of saliva collection (n)^*^	
Morning (8:50 am to 11:58 am)	54 (37.5)
Afternoon (12:00 pm to 5:07 pm)	90 (62.5)
Condition of gums (n)^†^	
Excellent	20 (13.9)
Very Good	43 (30.0)
Good	53 (36.8)
Fair	25 (17.4)
Poor	3 (2.1)
Has a loose tooth (n)^†^	5 (3.5)
IL-6 (pg/ml)^‡^	1.8 (0.61–6.8)
IL-8 (pg/ml)^‡^	54.6 (22.2–118.3)
IL-10 (pg/ml)^‡^	6.9 (2.6–18.6)
IL-1β (pg/ml)^‡^	1.1 (0.27–4.7)
TNF-α (pg/ml)^‡^	0.25 (0.13–0.50)
MMP-3 (pg/ml)^‡^ ^,^ ^‖^	197.5 (77.3–377.0)
MMP-8 (ng/ml)^‡^ ^,^ ^‖^	84.1 (35.5–183.6)
MMP-9 (ng/ml)^‡^ ^,^ ^‖^	305.0 (140.4–680.7)

*Data presented as mean ±SD.

†Data presented as number (%).

‡Data presented as geometric mean (25^th^-75^th^ percentile).

§N = 95.

‖N = 135.

### Principal components analysis

Table 2 presents the 5-component solution derived from the PCA. MMP-8 and MMP-9 were positively correlated with component 1 with factor loads of 0.89 and 0.88, respectively. IL-6, IL-1β, and IL-8 (factor loads of 0.82, 0.70, and 0.63, respectively) were highly correlated with component 2. Components 3, 4, and 5 were correlated with single analytes, TNF-α (factor load of 0.84), IL-10 (factor load of 0.87), and MMP-3 (factor load of 0.79), respectively. Subjects with high values on any or all of these components would also have high values for the respective markers that loaded highly on the component.

**Table 2:** Principal components analysis with orthogonal rotation of the individual cytokines and MMPs.

Component 1		Component 2		Component 3		Component 4		Component 5	
Marker	Load*	Marker	Load*	Marker	Load*	Marker	Load*	Marker	Load*
MMP-8	0.89^†^	IL-6	0.82^†^	TNF-α	0.84^†^	IL-10	0.87^†^	MMP-3	0.79^†^
MMP-9	0.88^†^	IL-1β	0.70^†^	IL-1β	0.40	IL-6	0.27	IL-6	0.34
IL-8	0.49	IL-8	0.63^†^	IL-8	0.35	TNF-α	0.26	MMP-9	0.24
IL-1β	0.44	TNF-α	0.35	IL-10	0.24	IL-8	0.26	MMP-8	0.19
MMP-3	0.39	MMP-3	0.33	MMP-3	0.21	MMP-3	0.22	IL-10	0.19
IL-10	0.25	IL-10	0.28	IL-6	0.19	MMP-9	0.20	TNF-α	0.19
TNF-α	0.23	MMP-8	0.26	MMP-8	0.19	MMP-8	0.17	IL-8	0.15
IL-6	0.16	MMP-9	0.22	MMP-9	0.15	IL-1β	0.15	IL-1β	0.11

*Factor loads are determined by the pearson correlation coefficient of the marker on the component.

^†^Factor loads >0.6 were considered for the interpretation of the component.

### Multiple linear and logistic regression model of PCA components

Table 3 presents the results of the multiple linear regression model of the PCA components on HbA_1c_, adjusted for age, sex, race, BMI, duration of diabetes, and gingival condition. In this model, a significant linear association was found between PCA component 1 (MMP-8 and MMP-9 loaded highly) and PCA component 3 (TNF-α loaded highly) with HbA_1c_ (β 0.28 ± 0.14, p = 0.045; β 0.31 ± 0.14, p = 0.029; respectively). The other PCA components were not found to be linearly associated with HbA_1c_ (p>0.05).

**Table 3:** Multiple^*^ linear regression of PCA components on HbA1c.

Marker	β ±SE	p-value
PCA Component 1 (MMP-8 & MMP-9)	0.28 ±0.14	0.045
PCA Component 2 (IL-6, IL-1β, & IL-8)	-0.02 ±0.14	0.901
PCA Component 3 (TNF-α)	0.31 ±0.14	0.029
PCA Component 4 (IL-10)	-0.11 ±0.14	0.421
PCA Component 5 (MMP-3)	0.21 ±0.14	0.123

^*^Adjusted for age, duration, BMI, sex, and gingival condition.

Table 4 summarizes the results of the multiple logistic regression model of the PCA components on the self-reported gingival condition. PCA component 2 (in which IL-6, IL-1β, and IL-8 loaded highly) was significantly associated with poorer gingival condition (OR 1.60, 95% CI 1.09–2.34; p-value 0.016) after adjustment for age, duration of diabetes, HbA_1c_, BMI, and sex. This result suggests that high values for these markers is associated with decreased gingival condition. None of the other PCA components was significantly associated with gingival condition (p>0.05).

**Table 4:** Multiple^*^ logistic regression of PCA components on condition of gums (poor + fair + good vs. very good + excellent).

Marker	OR (95% CI)	p-value
PCA Component 1 (MMP-8 & MMP-9)	1.16 (0.79, 1.70)	0.463
PCA Component 2 (IL-6, IL-1β, & IL-8)	1.60 (1.09, 2.34)	0.016
PCA Component 3 (TNF-α)	0.75 (0.52, 1.10)	0.141
PCA Component 4 (IL-10)	1.07 (0.74, 1.54)	0.717
PCA Component 5 (MMP-3)	1.05 (0.72, 1.53)	0.798

^*^Adjusted for age, duration of diabetes, HbA1c, BMI, sex.

## Discussion

This study demonstrated that specific salivary inflammatory markers in T1D subjects are associated with decreased glycemic control. Two principal components were associated with decreased glycemic control. The inflammatory markers that loaded strongly on these components were MMP-8, MMP-9, and TNF-α. This is the first study that we are aware of to examine the association of multiple salivary inflammatory biomarkers with glycemic control and self-reported gingival condition in T1D subjects.

Prior studies examining salivary inflammatory levels within general systemic diseases, T1D, and type 2 diabetes (T2D) have demonstrated that specific mediators of inflammation are elevated within the saliva of these respective cohorts. A large study examining the salivary concentrations of 1000 adults in southern Sweden for levels of IL-1β, -6, -8, TNF-α, and MMP-8 (similar to inflammatory markers examined in this study) measured increased levels of: (1) IL-8 in subjects with tumor and bowel diseases, (2) MMP-8 in those following cardiac surgery or with diabetes and muscle diseases and (3) IL-1β, -8, and MMP-8 in those having muscle or joint diseases [16]. With regard to salivary inflammatory burden in T1D, Dakovic *et al*. examined IL-8 salivary levels in T1D subjects with or without concomitant periodontitis versus the non-T1D cohort (n = 20 per group) [21]. Measured levels of IL-8 were significantly elevated in T1D subjects as compared to the non-T1D group. Within the T1D cohort, IL-8 levels were not associated with either periodontitis or clinical parameters. In respect to T2D, Yoon *et al*. examined unstimulated saliva samples in 192 subjects with or without T2D and revealed that IL-1β concentration in saliva was mainly associated with the degree of periodontal disease not diabetes [10]. Another investigation demonstrated that poor glycemic control (HbA_1c_ > 8) was significantly associated with increased IL-1β levels in gingival crevicular fluid in T2D [22]. In a later report, IL-8 levels did not associate with increased HbA_1c_ [23]. Taken together, these findings solidify the central hypothesis that salivary inflammatory burden can be associated with diseases of autoimmunity, metabolic control and periodontitis. However, the specific relationship between certain cytokines such as IL-1β and either glycemic or periodontal status can be contradictory and requires further characterization.

Proteomic and peptidomic analysis has revealed significant differences in the saliva between those subjects with T1D and periodontitis versus those with T1D and without periodontitis [12–14]. Concordantly, a recent study evaluating 153 subjects with T1D or T2D examined the proteomic profile of these individuals stratified by their HbA_1c_ levels ranging from <7 to >10 [24]. PCA and cluster analysis revealed that salivary proteomic changes were associated with HbA_1c_ sub-groupings and to some extent supported our findings in that systemic glycemic levels are reflected within the salivary milieu in an HbA_1c_ dependent manner. Their findings revealed that salivary proteomes are distinctly segregated when compared with low (HbA_1c_ <7), medium (8–9), and high (>10) HbA_1c_ levels. The proteomic changes based on HbA_1c_ were stronger in T1D rather than T2D subjects and the identified salivary proteins associated with HbA_1c_ changes in individual samples included albumin, hemoglobin, alpha-2-macroglobin, serum amyloid A, sereotransferrin, and numerous others. Interestingly, neither cytokines nor MMP’s were identified within the salivary proteome as associated with HbA_1c_ within T1D. This may have been attributed to a sensitivity issue whereby these inflammatory mediators are potentially below the detection level for this proteomic approach. There was no mention of periodontal status within this study. Nonetheless, their findings in combination with our report clearly demonstrate that glycemic levels as reflected by HbA_1c_ can be associated and represented in the saliva as measured by various biomarkers that can originate from the salivary gland, serum, or host immune system.

A recent report by Engebretson *et al*. revealed that periodontal intervention (non-surgical) failed to promote glycemic control in T2D subjects displaying moderate to advanced chronic periodontitis [25]. These findings would be somewhat discordant with our conclusions indicating that increased inflammatory burden is association with decreased glycemic control. However, systematic reviews and meta-analysis of numerous studies examining this relationship have indicated that periodontal treatment can result in modest reduction of HbA1c in combination with improvement of periodontal status in T2D subjects [26–29]. This type of comprehensive analysis has yet to be as extensively examined in T1D subjects with varied periodontal status as this was the primary cohort of our described study. Nonetheless, our results are certainly consistent with the overall theme that oral inflammatory levels are associated with glycemic control and potentially autoimmunity in T1D.

A potential weakness of our study is the lack of a non-T1D control group. The primary focus of this study was to measure and determine the association of salivary inflammation with glycemic control within T1D. Since glycemic control is an important component of T1D clinical management, but not for those without diabetes, we felt that this population was particularly relevant for this initial study. Previous literature has examined the gross comparison of salivary inflammation between diabetic (T1D and T2D) and non-diabetic controls and therefore, we did not intend to repeat these examinations [10, 21]. While we are not able to perform a comparison with non-diabetic controls, we are able to demonstrate that salivary inflammatory markers are significantly associated with increasing HbA_1c_ in a linear model, after adjustment for potential confounders.

Another potential limitation of this study is absence of a clinical dental exam with recorded measurement of pocket depth and bleeding on probing. Unfortunately, we were unable to perform a clinical exam due to both economic and logistical reasons. However, the primary objective of this study was to measure and examine quantitative measures of salivary inflammation with glycemic status in a T1D cohort. Clinical measures of periodontitis and periodontal inflammation obtained from an examination is strictly a qualitative determination of the inflammatory response. For our analysis, clinical measurements would certainly have provided some utility but would not have provided the quantitative assessment needed for our objectives. Nonetheless, we still accounted for this via self-reported gingival condition. Measurement of inflammatory mediators in the saliva provides a more comprehensive analysis of oral inflammation [11]. In addition, the inflammatory mediators utilized within this study that had the greatest association with increased HbA_1c_ levels (TNF-α, MMP8 and MMP9) have all been previously documented to be increased and associated with decreased periodontal status [17, 30, 31]. Therefore, it is reasonable to speculate that those individuals with elevated inflammatory burden also potentially had decreased periodontal health. Future studies are anticipated to include a comprehensive dental exam in combination with measurement of inflammatory burden and glycemic status.

Salivary diagnostics has tremendous translational potential for numerous biological and technical reasons. The outstanding utility of the saliva for serving as a clinical focal point during routine dental examinations or physician visits and potentially enabling large investigational studies certainly warrants this effort. As compared to blood collection, salivary evaluation is relatively easy to obtain with high patient compliance (in our study only 1% of subjects declined participation) and can be performed by minimally trained personnel with little post-collection processing. Our study has suggested that the salivary inflammatory burden may also be an indication of glycemic status or clinical management in T1D. These data can be further utilized to establish novel clinical diagnostic tools to promote patient compliance and enhance subsequent clinical application of interventional therapy. In addition, oral inflammatory burden with biomarkers described in this publication may also be combined with other biomarkers (ie. autoantibodies in the case of T1D) either circulating or from the saliva that can be implemented to generate predictive models to identify subjects in the early stages of either developing autoimmunity or glucose intolerance.
